# Population Dynamics of Six Major Insect Pests During Multiple Crop Growing Seasons in Northwestern New Mexico

**DOI:** 10.3390/insects10110369

**Published:** 2019-10-24

**Authors:** Koffi Djaman, Charles Higgins, Michael O’Neill, Shantel Begay, Komlan Koudahe, Samuel Allen

**Affiliations:** 1 Department of Plant and Environmental Sciences, Agricultural Science Center at Farmington, New Mexico State University, P.O. Box 1018, Farmington, NM 87499, USA; mmoneil@nmsu.edu (M.O.); samallen@nmsu.edu (S.A.); 2 Higgins Farms Inc., 4220 N. Crescent Ave, Farmington, NM 87401, USA; higginsfarms@comcast.net; 3 Wilbur-Ellis Co., 9813 NM-Hwy 371, PO Box 5370, Farmington, NM 87401, USA; SSBegay@wilburellis.com; 4 ADA Consulting Africa, 07 BP 14284, Lomé, Togo; kkoudahe_komlan@yahoo.fr

**Keywords:** fall armyworm, cabbage looper, corn earworm, beet armyworm, potato psyllid, western bean cutworm, seasonal abundance

## Abstract

This study was conducted to monitor the population dynamics of six major insect pests at the NMSU Agricultural Science Center at Farmington (ASC-Farmington) and within an adjacent commercial farm (Navajo Agricultural Products Industry, NAPI) for more effective and efficient pest management during the 2013–2019 period. Specific pheromone traps, sticky and net traps were used to collects moths of beet armyworm (*Spodoptera exigua*), cabbage looper (*Trichoplusia ni*), corn earworm (*Helicoverpa zea*), fall armyworm (*Spodoptera frugiperda*), potato psyllid (*Bactericera cockerelli*), and western bean cutworm (*Striacosta albicosta*). These insects generally appear in early June and their population decreases toward the end of August/early September with different peak times and magnitudes during July and August. *Bactericera cockerelli* was not substantially present in the commercial farm due to intensive insecticide application. Overall, all six insect species were present at ASC-Farmington, with relative abundance, in percent of the total collected moths by all traps, varying from 6.5 to 19% for *Trichoplusia ni*, 16 to 29.2% for *Spodoptera exigua*, 1.5 to 20.6% for *Striacosta albicosta*, 10 to 25% for *Helicoverpa zea*, 18.5 to 25.6% for *Spodoptera frugiperda* and 8.5 to 26.9% for *Bactericera cockerelli*. In NAPI’s commercial field, while the potato psyllid *Bactericera cockerelli* was not recorded, *Trichoplusia ni* and *Spodoptera exigua* showed decreasing rates that varied from 27.5 to 4.2% and from 49.3 to 7.8%, respectively. *Striacosta albicosta,* *Helicoverpa zea* and *Spodoptera exigua* showed increasing rates varying from 2.9 to 28%, from 7.8 to 25.3% and from 10.9 to 52%, respectively. The results of this study could serve as a guideline for sustainable management strategies for each of the six species for production profitability.

## 1. Introduction

In the effort to produce enough food and fiber for the increasing world population, many actions have been taken in the intensification of agriculture involving increasing trends of applied pesticides and efficient use of resources. World total pesticide use increased from 2.29 to 4.09 million tons during the 1990–2016 period [1]. Among the crop pests, insect pests cause incredible losses from planting to postharvest. Total annual pesticide used in the United States averages over 406,000 tons and insecticide represents about 21% of the total pesticide usage. Annual insecticide used in the US showed a slightly decreasing trend that average 86,727 tons per year during the 1990–2012 period [1]. Approximately 45 million ha were treated for insect damage across the USA in 2017 [2] and the damage caused by insect pests is still one of the major causes of production losses and quality deterioration [3]. While numerous efforts are made to control insect pests, the environmental impact of insecticide applications is alarming in terms of the direct effects of human consumption, residual concentration in the agroecosystem, contamination of soil and water, and development of resistance by insect pests.

Insect pest abundance and population dynamics are affected by abiotic and biotic stresses impacting insect morphology, physiology, and their adaptation to the environment [4,5]. Danks [6] indicated that climatic suitability for insect development and its population dynamics directly influences habitat suitability. Régnière et al. [7] reported that high temperature threshold is the main factor governing insect population dynamics. Shatz et al. [8] found that various biotic and abiotic factors were responsible for the distribution of Asian Longhorn Beetle (*Anoplophora glabripennis*) across Worcester County, Massachusetts (USA). Finlay-Doney and Walter [9] reported that temperature can stimulate or suppress insect pest genetic potential, mortality and fecundity, and host plants. Danks [10] studied the elements of seasonal adaptation in insects and found the air temperature, air relative humidity, aridity, food availability, sensitivity of environmental signals, interaction among insect species and other factors as responsible elements controlling insect abundance [11,12,13,14].

Crop production in the northwestern region of the state of New Mexico is mainly dominated by the industrial agri-business company Navajo Agricultural Products Industry (NAPI), which has developed more than 30,000 ha of irrigated cropland, equipped with center pivot irrigation systems and producing farm crops such as alfalfa, corn, small grains, potatoes, beans, and pumpkins, with the potential to develop an additional 15,000 ha. The most dominant insect pests found in the region on alfalfa, beans, corn, peppers, potatoes, tomato, wheat and other crops are beet armyworm (*Spodoptera exigua*), cabbage looper (*Trichoplusia ni*), corn earworm (*Helicoverpa zea*), fall armyworm (*Spodoptera frugiperda*), potato psyllid (*Bactericera cockerelli*), and western bean cutworm (*Striacosta albicosta*) and NAPI has been using integrated pest management approaches.

The monitoring of the pest population and the identification of the prevailing insects will allow for timely application, using the best methods and applied rates relative to the economic threshold of each insect pest. It is, therefore, important to study pest population dynamics during the crop growing season and create a benchmark that could be used by crop producers, ecologists, agricultural economists, researchers and consultants for efficient and effective insect pest control. The objective of this study was to monitor the temporal abundance of six major insect pests during multiple cropping seasons in order to help crop producers of the region achieve efficient, sustainable and environmentally sound use of insecticides.

## 2. Materials and Methods

**Figure 1:**
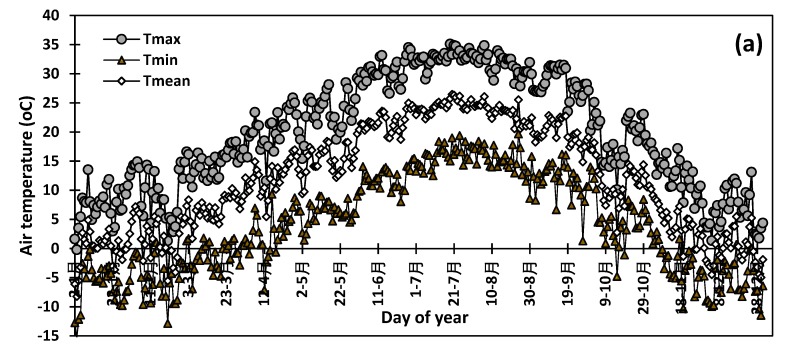

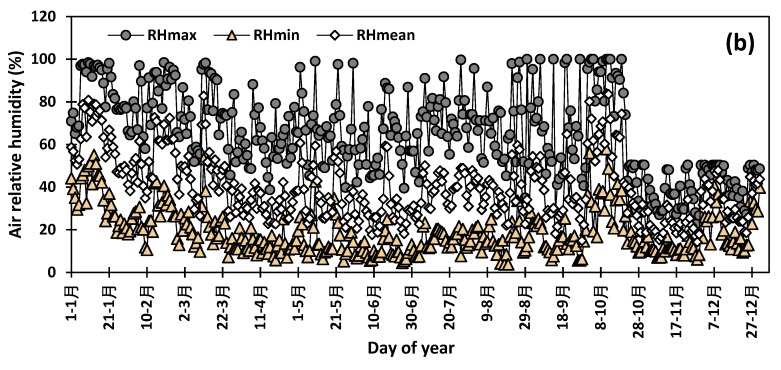
Average air temperature (**a**) and average air relative humidity during the study period (**b**).

The current study was conducted at the New Mexico State University (NMSU) Agricultural Science Center at Farmington (Latitude 36.69′ North, Longitude 108.31′ West, Elevation 1720 m). It was initiated in spring 2016, following initial monitoring of insect counts at ASC-Farmington and Navajo Agricultural Products Industry (NAPI) starting in 2013. NMSU Agricultural Science Center is located within the NAPI farm and a part of this study was conducted in NAPI’s plots in 2016 and 2017 growing seasons. Weather variables were monitored at the experimental station by an automated weather station. The average 2013–2018 period minimum temperature (Tmin), maximum temperature (Tmax), average temperature (Tmean), minimum relative humidity (RHmin), maximum relative humidity (RHmax), and average relative humidity (RHmean) are presented in Figure 1.

**Figure 2:**
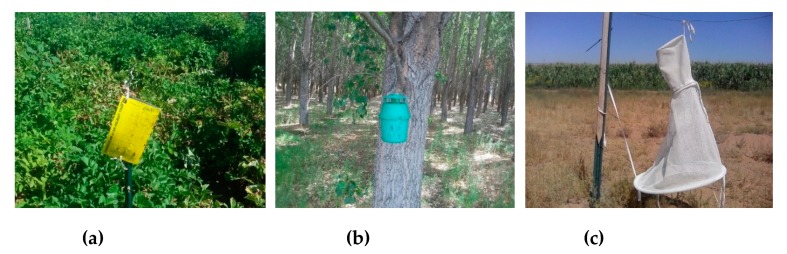
Sticky trap (**a**), bucket/funnel trap (**b**) and net trap (**c**) used to collect moths.

Insect traps used for the current study consisted of bucket/funnel traps (Great Lakes IPM) (Vestaburg, MI, USA), nylon mesh heliothis/Hartstack traps (Scentry, Great Lakes IPM), sticky traps (Trécé Pherocon AM) (Trécé Inc., Adair, OK, USA) and delta traps (Trécé Pherocon VI). Aside from sticky traps specific for potato psyllid monitoring, each trap was re-baited on a weekly basis with species-specific pheromone caps (Trécé Pherocon) related to each of the other insect pests (*Spodoptera frugiperda, Helicoverpa zea, Striacosta albicosta, Spodoptera exigua, and Trichoplusia ni*) to lure male moths into the trap. The pheromone caps were purchased early April and stored in a refrigerator at the research station. They were carefully handled to avoid any contamination from each other. They were double checked always before being placed in the traps and, to avoid any contamination the name of specific insect pest was written on the bucket. Among the various types of traps, sticky traps were used for direct monitoring of potato psyllids (*Bactericera cockerelli*) in the potato fields wherein 5 traps per field, evenly spaced in one block running N/S were installed at a height of about 1 m (Figure 2a). At the same alignment of the potato plots, were some sweet corn, hybrid Sorghum-Sudan grass plots, peanut, and other fallow plots. Adjacent to these plots is a 2007 hybrid poplar planted plot covering 2.75 ha. The pheromone bucket traps were placed in a nearby line of hybrid poplar trees (*Populus* spp.) (Figure 2b) at 1.5–2 m height adjacent to fields about 18 m from the potato fields. The net traps were mounted on a two-meter metallic bar and were placed about 10 m away from the edge of center pivot irrigated corn field at the height of approximately 0.8 m. Tent nets were used for corn earworm (*Helicoverpa zea*) (Figure 2c), and bucket/funnel traps were used for other potential insect pests: Beet armyworm (*Spodoptera exigua*), cabbage looper (*Trichoplusia ni*), fall armyworm (*Spodoptera frugiperda*), potato psyllid (*Bactericera cockerelli*), and western bean cutworm (*Striacosta albicosta*). Visual monitoring of insect damage to agricultural crops was conducted weekly. This included psyllid yellow and or presence of potato psyllid nymphs at the underside of potato leaves in potatoes and feeding damage on young leaves of corn, feeding tunnels in the stalks and corn ears, moist castings at the end of on corn ears in a nearby corn field (data not reported). Insect counts of each species were tallied weekly. The catch of each bucket trap and net trap was transferred into a plastic ziploc® bags and brought to the lab for counting and identification. Incidence of large numbers of a particular insect species, was used in decision-making on need for insecticide application at NAPI. Data were collected during the active growing season, generally from late May/early June through late August/September.

Data were analyzed using CoStat statistical software. Three-way ANOVA was performed with the main effects as species (six species under this study), year (2013 to 2018) and date (two-week interval from June 1st to September 15) and their interactions. All *p* values ≤ 0.05 were considered statistically significant. The means were separated using Fisher’s protected least significance difference (LSD) test at the 95% level of probability to identify significant differences between the treatments.

## 3. Results

### 3.1. Variation in the Abundance of the Six Insect Species

**Figure 3:**
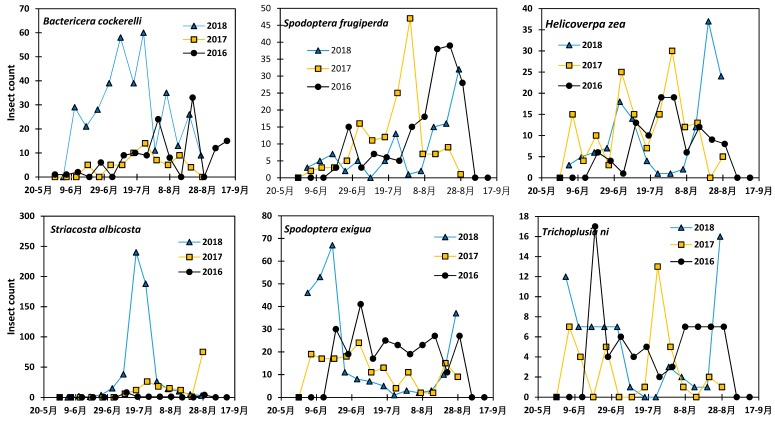
Variation in the population density of six insect pests at New Mexico State University Agricultural Science Center at Farmington during the 2016–2018 cropping periods.

The variation in the abundance of the six insects under this study during crop growing season for the 2016–2018 period is presented in Figure 3. While the year, pest species and the date of moth collection did not have significant effect on the moth abundance, the interactions year - species (*p* = 0.020) and year - date (*p* = 0.011) are significant effect on the pest abundance (Table 1). However, more insects were collected during the 2015 growing season, followed by 2018, 2016, 2017, 2013 and 2014 (LSD = 14). *Bactericera cockerelli* was the most abundant pest with an average of 29 adults captured and *Trichoplusia ni* was the least abundant pest with an average of eight adults captured on a two-week basis. Overall, insect pests were more present during the second half of July and they were the least present in early June (LSD = 15.45). There was non-significant inter-annual variation in the peak time of each of the insects. At the research station, the number of collected moths increased with the growing season and decreased toward the end of the growing season. The population of *Bactericera cockerelli* showed two peaks on 1 and 22 August 2016, and 24 July and 14 August 2017. During the 2018 crop growing season, *Bactericera cockerelli* still showed two peaks on 9 July and 23 July. There were less *Bactericera cockerelli* moths in 2017 compared to 2016 and 2018. *Spodoptera frugiperda* showed a peaks in the number of individuals in the population on 22 August, 31 July and 29 August in 2016, 2017 and 2018, respectively. *Helicoverpa zea* showed more than one peak during the growing season during the 2016–2018 period with similar abundance during the three growing seasons. There were huge differences in *Striacosta albicosta* number among the three growing seasons and the highest peak of 240 moths collected in 2018 while the peaks were 6 in 2016 and 75 in 2017 after a previous lower peak of 26 on 24 July 2017. However, the peaks appeared during the mid-season from mid to the end of July, usually corresponding to maize development, tasseling and silking stages. Overall, *Spodoptera exigua* was abundant during the 2016–2018 period with the highest peak of 41, 24, and 67 in 2016, 2017, and 2018, respectively. *Trichoplusia ni* showed similar distribution like *Spodoptera exigua* with different peaks during the growing seasons. However, the highest counts of 17, 13 and 16 moths were registered in 2016, 2017, and 2018, respectively.

**Figure 4:**
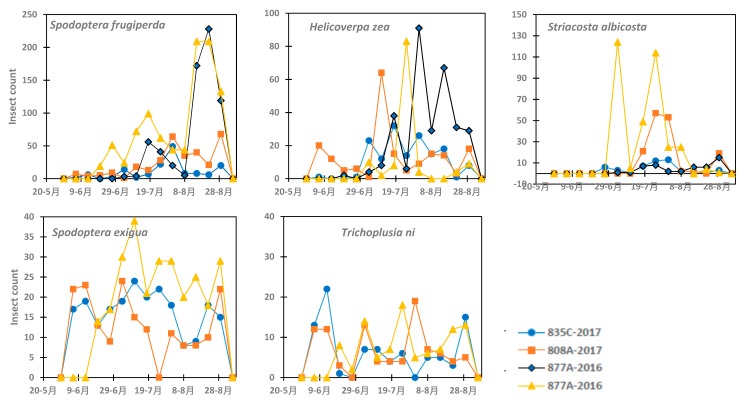
Variation in the population density of five insect pests at the Navajo Agricultural Products Industry farm (*Bactericera cockerelli* was not present).

In the commercial fields at NAPI, insect pests were more abundant in 2016 than in 2017 (Figure 4). *Bactericera cockerelli* was not collected during the 2016 and 2017. *Spodoptera frugiperda* peaked on August 22 and August 15–22 in 2016 in the two fields and on 31 July and 7 August 2017 in the other two fields with a second peak at the end of August 2017. *Helicoverpa zea* peaked late July 2016 and mid-July 2017. The peak population was 91 and 83 in both fields in 2016 while it was 32 and 64 in the two fields respectively in 2017. There was large variation between fields in terms of the abundance of *Striacosta albicosta* with a peak by the end of July in 2016 and 2017. However, it presented one first peak in one field on 4 July 2016. *Spodoptera exigua* was present only in one field in 2016 with the greatest peak of 39 during the growing season. It was present in both fields in 2017 with a peak of 24 moths in both fields on July 3 and 10, respectively. *Trichoplusia ni* was present in a lower number compared to the other species. The peak was 18 count in one field in 2016 and 22 and 19 in the two fields in 2017, respectively. *Trichoplusia ni* abundance peaked on July 25 in 2016 and June 29 and August 7 in the two fields, respectively, in 2017; however, it was always present during the growing season.

### 3.2. Monthly Distribution of the Six Insect Species at the Research Station for the 2013–2018 Period

**Figure 5:**
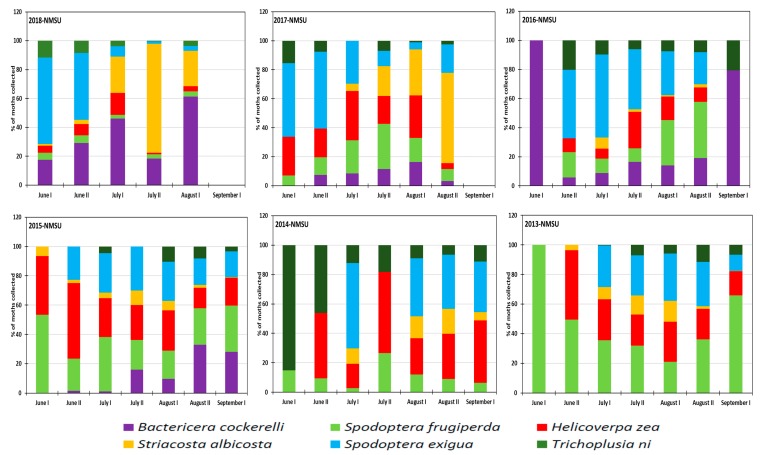
Proportions of insect species collected at the NMSU ag research station during the 2013–2018 period.

Abundance of the studied insects varied with growing seasons at the NMSU Agricultural Center as shown in Figure 5. *Trichoplusia ni* was less abundant compared to all other species in most seasons except in 2014, representing 85% of collected moths during the first half of June and 46% in the second half of June 2014. *Spodoptera frugiperda* was the most dominant throughout the 2013 growing season and was dominant during the period of July–August 2016 and 2017. It was the least recorded insect during the 2018 growing season at NMSU Science Center. *Bactericera cockerelli* was revealed to be the major insect pest at the beginning of the growing season and its population density diminished as the season progressed. It appeared during the first half of June 2017, the second half of June 2016, and 2015 while it appeared the first half of July 2013 and 2014. *Striacosta albicosta* was the least abundant species among all six species with a maximum annual rate of 14% in 2013, 17% in 2014, 10% in 2015, 8% in 2016 and a large rate of 62% in 2017 and 75% in 2018. The large rates of *Striacosta albicosta* in 2017 were recorded in late July while the large rate in 2018 was recorded late August. *Helicoverpa zea* was always present from June to late August with a peak density not occurring at the same period (Figure 5) as it was late June 2013 and 2015, late July 2014 and 2016 and early July in 2017 and 2018. It was less abundant during the 2018 growing season. *Spodoptera exigua* was revealed to be one of the major species, and which should be considered in terms of insecticide application in the study area. It represented a maximum of 32% in 2013, 58% in 2014, 30% in 2015, 56% in 2016, 53% in 2017 and 60% in 2018 at different periods between June and July during the growing season (Figure 5).

At NAPI field during the 2016 growing season, *Spodoptera frugiperda* was highly abundant with a rate varying from 30% to 80%, while it represented 11 to 60% of the collected insects in 2017. *Striacosta albicosta* was the second most dominant pest in 2016 while *Spodoptera frugiperda* was the second most abundant pest in 2017 with the highest rate occurring in June. *Trichoplusia ni* was the least abundant species in the commercial field with prevalence rate varying from 9% to 2% in 2016 and from 28% to 6% in 2017 with a diminishing rate as the growing season progressed. Similar behavior was observed for *Spodoptera exigua* with abundance rate varying from 27% to 6% in 2016 and from 49% to 10% in 2017. *Bactericera cockerelli* was not present across the NAPI commercial field where table and chip potatoes are grown at large scale with predetermined insecticide application schedule.

**Figure 6:**
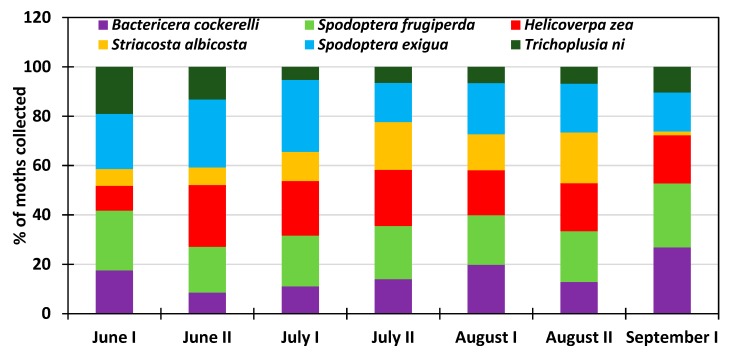
Average proportion of moths collected during the 2013–2018 period at NMSU Agricultural Center.

**Figure 7:**
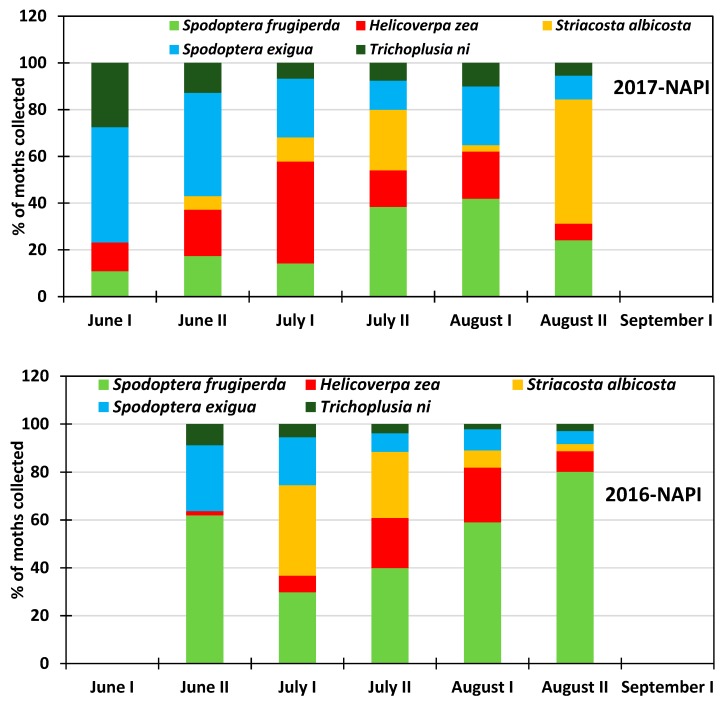
Proportions of insect species collected at the research station during the 2016–2017 period at NAPI commercial field.

**Figure 8:**
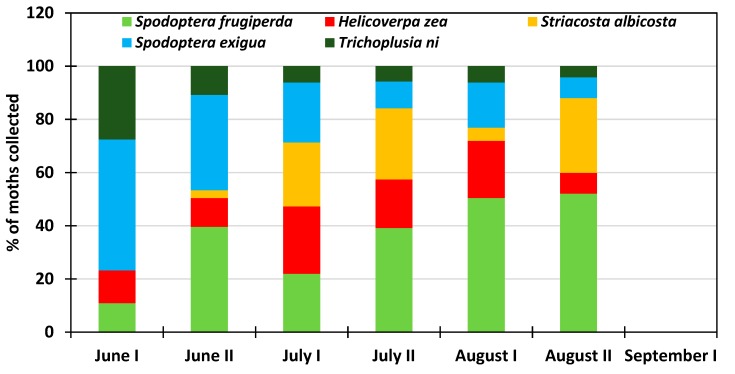
Average proportion of moths collected during the 2016–2017 period at NAPI commercial field.

All six insect species in this study were present at the NMSU Agricultural research station at Farmington (Figure 6). The abundance rate of *Trichoplusia ni* varied from 19% in early June to 6.5% late July and increased thereafter to 10.4% early September. *Spodoptera exigua* was consistently more abundant than *Trichoplusia ni* representing 16% to 29.2 % of the collected moths, and was more abundant in late June and early July. Among the studied species, *Striacosta albicosta* was the least abundant at the beginning of the growing season representing 7% in June; the peak of 20.6% was reached late August and the population rate dropped to 1.5% early September. *Helicoverpa zea* was also present throughout the growing season with a prevalence rate of 10% early June and varied from 25% to 18% during the rest of the growing season period. Like *Spodoptera exigua, Spodoptera frugiperda* was present at a relatively constant rate that varied from 18.5% to 25.6% and averaged 21.6% for the entire growing season. Overall, the potato psyllid *Bactericera cockerelli* was also present during the growing season. Its percent abundance rate varied from 8.5% to 26.9% and was more abundant at the end of the growing season. At NAPI’s commercial field, the potato psyllid was not recorded, probably due to specific insecticide (Actara, Sivanto Prime, Minecto Pro) application against this insect pest (Figure 7). *Trichoplusia ni* showed a decreasing rate that varied from 27.5% in early June to 4.2% in late August. Likewise, *Spodoptera exigua* also showed a decreasing trend from 49.3% early June to 7.8% late August. *Striacosta albicosta* appeared during the second half of June and its population rate increased from 2.9% to 28% with a reduced prevalence of 5% at the beginning of August. *Helicoverpa zea* was always recorded throughout the growing season with a rate that varied from 7.8% to 25.3% with the highest prevalence from July to early August corresponding to maize silking stage in the study area. *Spodoptera frugiperda* was overall the most abundant species with an increasing rate from 10.9% in early June to 52% late August (Figure 8).

**Figure 9:**
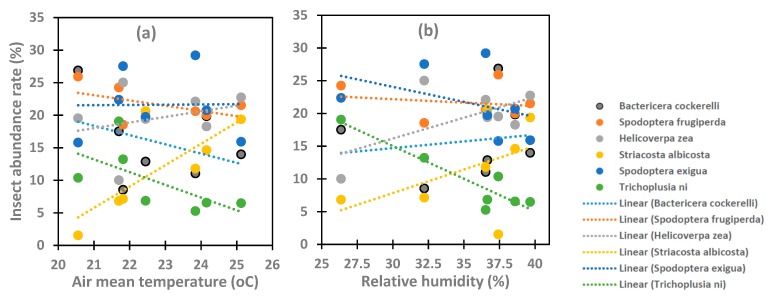
Correlation between insect abundance and (**a**) average air temperature and (**b**) air relative humidity for the study area.

Studied species showed different relationships with the average temperature and relative humidity (Figure 9). The population *Striacosta albicosta, Spodoptera exigua* and *Helicoverpa zea* increased with increasing average air temperature with coefficient of determination R^2^ values of 0.57, 0.001 and 0.08, respectively, while the population of *Bactericera cockerelli, Trichoplusia ni* and *Spodoptera frugiperda* decreased with increasing average air temperature with coefficient of determination R^2^ values of 0.14, 0.40 and 0.26 respectively. *Trichoplusia ni* showed a strong negative correlation with air relative humidity (R^2^ = 0.85). Silimarly the population of *Spodoptera exigua* (R^2^ = 0.16) and *Spodoptera frugiperda* R^2^ = 0.04) also showed negative correlation of their population abundance with the relative humidity. The population of *Bactericera cockerelli, Striacosta albicosta* and *Helicoverpa zea* showed positive correlation with air relative humidity.

## 4. Discussion

Understanding the population dynamics of the insect pests of the major crops grown in the northwestern New Mexico can provide important information that will aid in these pests’ management mostly within the large commercial farms. Different factors influence insect population temporal and spatial abundance. The six insect species under the present study appeared by the end of May early June during all crop growing season from 2013 to 2018. Across the northwestern New Mexico, average crop planting date is around mid-May and corn and dry beans are planted until mid-June. Pest presence and abundance magnitude presented in this study did not aligned with most similar studies. *Spodoptera frugiperda, Helicoverpa zea, Trichoplusia ni, Striacosta albicosta,* and *Spodoptera exigua* appeared sooner at different locations due the late establishment of crop growing season because of the high altitude with late spring frost [15]. *Trichoplusia* ni has two generations a year with prevailing moths from late April to mid-May and from late July to August in Oregon and British Columbia [16] and the specie is still present after August in Oregon [17], after September in British Columbia and November in California [18] with the last peak in August for most locations. Franklin et al. [16] indicated no presence of *Trichoplusia ni* in Yuma (Arizona) during the 2006 summer due to the lack of host crops and high temperatures while it was present in November on crucifer crops. Lafontaine and Poole [19] reported that *Trichoplusia ni* migrates north up to Canada each spring from the southern United States where it overwintered. Parajulee et al. [20] reported one population peak of *Helicoverpa zea* at Halfway and two peaks at Lubboock, Texas while adult moths are present from April to October. In Maryland, *Helicoverpa zea* is present from early May to early October and the population peaked of in late August and early September [21,22]. *Helicoverpa zea* was found in Lucerne in California with the highest population occurring late spring and late summer when the peak occurred early summer in cotton and October in lettuce [26]. Smith et al. [23] reported *Striacosta albicosta* highest abundance count in late July while the flight begins during the first week of June and continues to early September in the great lakes region from Michigan to Ontario [24]. The soil types at the study area is highly sandy soils [25] which might be favorable of *Striacosta albicosta* larvae deep overwintering [23,26]. The presence period of *Helicoverpa zea* and *of Striacosta albicosta* coincided with corn growing period as corn is planted in the study area from late April to early June and harvested in October for the first planting and in December for the late planting. Vajgand et al. [27] reported one population peak of *Spodoptera* exigua on 19 August 2016, while they recorded five population peaks from 9 July to 2 September 2003 in in Serbia. Debolt et al. [28] used light and pheromone traps to collect *Trichoplusia ni* moths near Red Rock (Arizona) and found that while the insect is present all year long, moths peak abundance occurred during August or September each year. Franklin et al. [16] found that *Trichoplusia ni* migrates northward and could reach British Columbia after overwintering in southern California, and collected *Trichoplusia ni* from May to November across the west from Arizona to British Columbia. *Trichoplusia ni* is unable to survive air temperatures lower than 10 °C and its populations are clustered between 35° and 40° latitudes in southern California and Mexico [29,30]. McGrath [17] reported that *Trichoplusia ni* is abundant late April mid-May, late July–August with population peak in August in Oregon. Further north in British Columbia, moths are collected after September and their numbers increase with availability of crops [17]. Allen and Luttrell [31] found maize planted area to influence *Helicoverpa zea* abundance in June than later in the growing season due to the distribution of the species across the agricultural landscape in southeast Arkansas. Late appearance of *Spodoptera exigua* was pointed by Adamczyk et al. [32] who reported that *Spodoptera exigua* moths start immigration in the western delta regions of Mississippi at mid-July using pheromone traps while Hendricks et al. [33] indicated that the highest abundance of the same pest occured in September–October in the lower Mississippi Delta while present in all months of the year. DiFonzo and Hammond [34] used pheromone traps and collected *Striacosta albicosta* moths at the beginning July in Ohio and from mid-July to August in Michigan who are also the northern states. Munyaneza et al. [35] indicated that *Bactericera cockerelli* appears late July with a peak by the end of August/early September at Moxe, Washington. As reported in this study, *Bactericera cockerelli* did not appear before early June in Washington with difference in colonization timing [35] whenever the potato plants started sprouting late April. Antolínez et al. [36] used sweep net Irwin traps and found *Bactericera trigonica* and *Bactericera nigricornis* on carrot and potato crops in Spain from crop emergence to harvest with peak population occurring from June to October related to field geographical locations. Teresani et al. [37] used sticky traps and reported different psyllid species existence in summer with population peak in August in Villena and July in Tenerife (Spain). Goolsby et al. [38] found inter-annual, seasonal, and spatial variation in the population of *Bactericera cockerelli* across the states of Colorado, Kansas, Nebraska and Texas during three-year experiment with the yellow sticky traps. Insect pest abundance was also affected by the weather conditions such as air temperature and relative humidity. Insect abundance showed different correlations with air temperature and relative humidity as reported by numerous studies. The findings of the present study are in agreement with Parajulee et al. [20], who reported significant positive correlation between air temperature, the thermal units, weekly average precipitation and *Helicoverpa zea* abundance during the period from June to September in the Texas High Plains region. Munyaneza et al. [35] reported that *Bactericera cockerelli* migrated biannually with high temperature and wind in late spring from western Texas to southern New Mexico, Arizona, California and northern Mexico [39,40,41]. Several studies reported the size of spring populations, wind patterns, air temperature and the presence of non-crop host plants are the factors that affect *Bactericera cockerelli* migration [39,40,41]. Cranshaw [40] and Capinera [41] reported that optimum temperature for the development of *Bactericera cockerelli* is about 27 °C with oviposition, hatching, and survival reduced at 32 °C and ceasing at 35 °C. In the study area, *Spodoptera frugiperda* has a strong impact on alfalfa, corn, beans, potatoes and other crops. Different vegetative landscapes across the study area might be habitats including non-crop hosts for the insect species [42,43]. González et al. [44] indicated that insect pest population dynamics may be attributed to the presence of the insect in non-crop hosts in the crop field environment with periodic movement between crop hosts and non-crop hosts. The insufficiency of the present study is the deepen evaluation of the correlation between pest abundance the weather parameters and the influence that the specificity pest-host plants might have on the temporal and spatial dynamics of the pest population across the northwestern New Mexico.

The population peak timing reported in this study for each of the six major insect pests in the northwestern New Mexico is useful for pest management. The population dynamics of the pests under this study may be used as guideline for insecticide application. *Spodoptera frugiperda* lays eggs on the green husks of corn ears and on the lower side of the leaves. The population peak of *Helicoverpa zea* coincides with corn silking stage while *Striacosta albicosta* hatching coincides with corn tasseling and silking stages. Crop producers must proceed with timely insecticide application to reduce the population of the pests below the economic threshold for a specific or all insect pests in the study area [23,26,45]. While economic threshold of about 14% equivalent to one pest egg mass per seven plants or 8% of plants with egg masses or very small larvae for the western USA [45,46] and three adults of potato psyllid per sticky trap [45], more research should be conducted locally to determine the economic thresholds for the insect pests under this study. Across NAPI farms, there are plots of potatoes, corn, dry beans, wheat, Sorghum-Sudan grass and these crops are hosts of several insect pests. Therefore, grouped insecticide application on different crops is recommended to avoid pest migration from one plot to another even when these plots have different host crops.

## 5. Conclusions

The study monitored population dynamics of six crop pests during six growing seasons in northwest New Mexico. While there was temporal variation in pest population from early June to early September, there was inter-annual variation in the moth abundance peak of the studied species. The results of this study could aid in crop pest management in the study area. However, there is a need for additional research to determine the non-crop hosts of the species and determine the economic threshold for better pesticide application for production profitability, conservation, and environmental sustainability of the insecticide application programs. This study may help in insecticide application timing using the appearance of specific insect pests on the crops and their population peak densities in consideration for economic management.

## Figures and Tables

**Table 1:** Summary of the analysis of variance of insect pest population dynamics (effect of factors or interaction between factors is significant if *p* value is lower than 0.05).

Source	df	Type III SS	MS	F	P	Significance
Year	5	8642.37	1728.47	1.569	0.1721	ns
Species	5	11723.69	2344.74	2.129	0.0650	ns
Date	6	14055.86	2342.64	2.127	0.0534	ns
Year * Species	25	48535.17	1941.41	1.763	0.0204	*
Year * Date	30	59535.60	1984.52	1.802	0.0116	*
Species * Date	30	34129.07	1137.64	1.033	0.4296	ns
Error	150	165207.04	1101.38			

Significance: ns = non-significant; * = significant at *p* value = 0.05.
